# Thyroid Storm Triggered by Strangulation in a Patient with Undiagnosed Graves' Disease

**DOI:** 10.1155/2018/4190629

**Published:** 2018-01-02

**Authors:** Jorge I. Conte, Marilyn A. Arosemena, Kunal Kapoor, Naomi G. Dempsey, Megan L. Zaleski, Atil Y. Kargi

**Affiliations:** ^1^Department of Medicine, Jackson Memorial Hospital/University of Miami, Miami, FL, USA; ^2^Division of Endocrinology, Department of Medicine, University of Miami Miller School of Medicine, Miami, FL, USA

## Abstract

Thyroid storm is the life-threatening end-organ manifestation of severe thyrotoxicosis. If left untreated, thyroid storm may cause acute heart failure, multiorgan dysfunction, and death. A high degree of suspicion is necessary to make the diagnosis and start antithyroid medications to decrease mortality. Thyroid storm is generally seen in patients with Graves' disease but should also be suspected in patients with fever, tachycardia, altered mental status, and risk factors including local trauma to the neck, such as strangulation. Based on our review, we report the first case of thyroid storm after strangulation as the presentation of previously undiagnosed Graves' disease.

## 1. Introduction

Thyroid storm (accelerated hyperthyroidism) is a rare, life-threatening syndrome characterized by end-organ compromise most commonly occurring in patients with known history of Graves' disease but may also be the initial presentation of thyrotoxicosis. Less commonly, thyroid storm may be precipitated by an acute event, such as surgery, infection, diabetic ketoacidosis, or parturition [1–4]. Blunt force, penetrating trauma, and suicide attempt by hanging have also been reported as precipitants of thyroid storm. Only one case of strangulation induced-thyroid storm has been reported that we are aware of; however unlike our patient, that patient had no underlying thyroid disease [5–9].

## 2. Case Presentation

**Figure 1:**
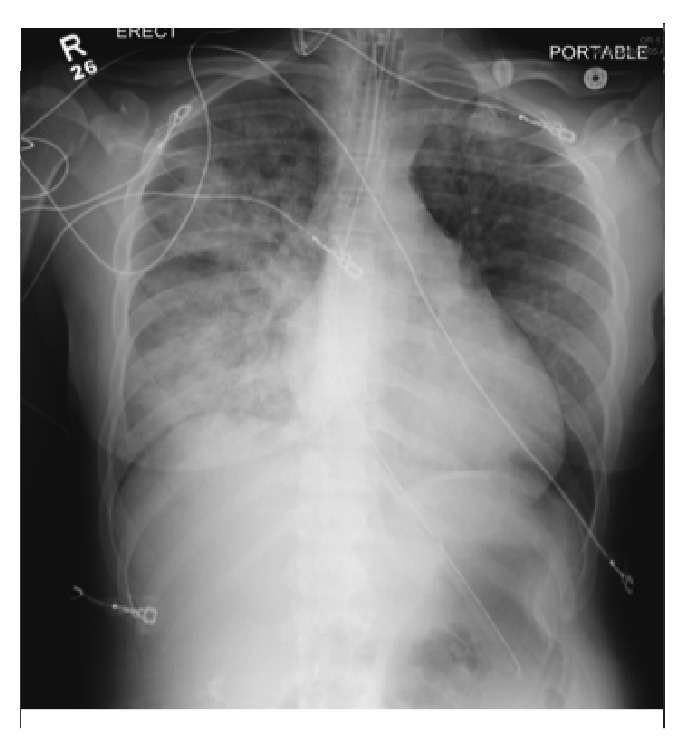
Chest X-ray: pulmonary edema with predominant right side opacities.

A 32-year-old African American female without significant past medical history presented to the emergency department with shortness of breath after being sexually assaulted and strangled. On arrival, her heart rate was 160 beats/minute, blood pressure 120/70 mmHg, respiratory rate 50 respirations/minute, and oxygen saturation 80%. She was alert and oriented but severely agitated and in respiratory distress with proptosis, ocular chemosis, and soft tissue swelling of the neck noted on physical exam. An EKG showed sinus tachycardia. Echocardiogram showed an ejection fraction of approximately 47% with regional wall hypokinesis, likely representing stress cardiomyopathy. Chest X-ray revealed predominantly right-sided pulmonary opacities, suggestive of pulmonary edema (Figure 1), and she required intubation due to persistent hypoxia with noninvasive measures.

**Figure 2:**
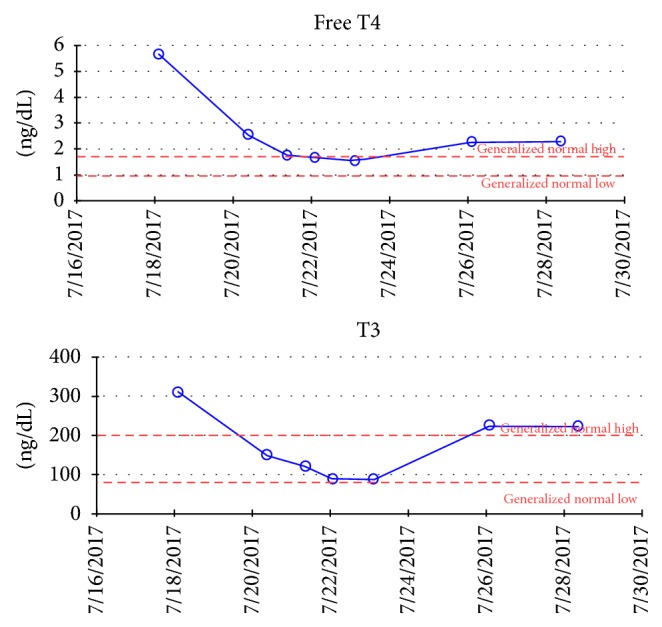
Free T4 and T3 levels.

The next day, her temperature reached 40.6°C and remained elevated despite acetaminophen, ibuprofen, and ice packs. Her tachycardia of up to 180 beats/minute persisted despite fluid resuscitation and beta blockade. Due to the combination of persistent hyperpyrexia, tachycardia, pulmonary edema, altered mental status off sedation, and proptosis, thyroid storm was suspected. Thyroid function testing revealed thyrotoxicosis with T4 > T3 (T4 dominant thyrotoxicosis) and suppressed TSH. Laboratory values were fT4: 5.66 ng/dl (normal range, 0.93–1.70), T3: 309.9 ng/dl (normal range, 80–200 ng/dL), and TSH < 0.005 mcIU/mL (normal range, 0.270–4.200 mcIU/mL) (Figure 2). A Burch-Wartofsky score was calculated on day two with a score of 75 (temperature +15, agitation +10, absent gastrointestinal symptoms 0, tachycardia +25, pulmonary edema +15, and precipitating event +10) highly suggestive of thyroid storm. The patient was also classified as a definite case by the Japanese Thyroid Association diagnostic criteria and she was started on treatment with methimazole 60 mg daily through nasogastric tube, stress dose hydrocortisone 50 mg IV every 6 hours, and propranolol 60 mg every 4 hours through nasogastric tube.

Two days later, thyroid hormones decreased significantly with T3 149.4 ng/dl and FT4 2.54 ng/dl. Additionally, her temperature, heart rate, pulmonary edema, and mental status gradually improved. On day ten, after five days of normal thyroid hormones, methimazole dose was decreased, hydrocortisone was discontinued, and propranolol was titrated off; however there was a slight increase in the FT4 and T3 which can be appreciated in Figure 2, and the dose was readjusted and she was extubated and safely left the ICU.

Prior to discharge, the patient was questioned about symptoms of hyperthyroidism before the assault. She endorsed palpitations but otherwise denied prior symptoms of hyperthyroidism. Furthermore, she denied personal or family history of autoimmunity. Laboratory findings showed positive TSH receptor antibody at 53.5% inhibition (normal range, <16% inhibition) confirming her diagnosis of Graves' disease. The patient was discharged on day fourteen with follow-up in the endocrinology clinic.

## 3. Discussion

Trauma is a rare precipitant of thyroid storm, accounting for 3.9% of cases in a nationwide survey of Japanese hospitals [9]. Hypotheses for thyroid storm due to trauma include a release of preformed thyroid hormone due to ruptured acini, increased responsiveness to catecholamines, and enhanced cellular responses to thyroid hormone [1–4, 6, 8].

The diagnosis of thyroid storm is based upon the presence of severe, life-threatening signs and symptoms (tachycardia >140 beats/minute, hyperpyrexia of 40-41°C, and central nervous systems symptoms such as agitation, anxiety, delirium, or psychosis) in a patient with biochemical evidence of hyperthyroidism. Of note, the serum thyroid hormone level is not significantly greater than those in severe uncomplicated thyrotoxicosis and therefore serum levels alone are not used to diagnose thyroid storm. It is the inability to adapt to the metabolic stress that results in thyroid storm. In our patient, resistant tachycardia to 180 beats/minute and fever to 40.6°C in combination with biochemical evidence of hyperthyroidism raised high suspicion for thyroid storm [1–4].

Diagnosing thyroid storm in the ICU can be challenging due to coexistence of fever and distributive shock that may be present in critically ill patients with overlapping syndromes such as sepsis. In our patient, prior history of strangulation, negative blood cultures and unresponsiveness to fluid resuscitation and antibiotics made sepsis less likely. The diagnosis of thyroid storm was confirmed once clinical and biochemical improvement was noted within 24–48 hours of the administration of antithyroidal medications.

There are no universally accepted criteria or validated clinical tools for diagnosing thyroid storm. However Burch and Wartofsky introduced a scoring system using clinical criteria, with a score ≥45 being highly suggestive of thyroid storm. Our patient had a score 75 making thyroid storm very likely [3, 5, 7].

Per literature review, there are only 25 mentioned cases of trauma-induced-thyroid storm, all of which, save one, were secondary to motor vehicle accidents or trauma other than strangulation. Most cases occurred in young women with median age of 32 years [9]. From those cases, nearly 50% had history of hyperthyroidism with or without underlying treatment. There was clinical suspicion of Graves' disease in our patient on presentation based on her young age, evidence of exophthalmos, and diffusely enlarged gland; however, strangulation itself may also cause exophthalmos and swelling of the neck from venous congestion. Nonetheless, the diagnosis of Graves' disease was confirmed in our patient with positive antibodies and this underlying condition increased her risk of developing thyroid storm after trauma [5].

The outcomes of thyroid storm largely depend on immediate and appropriate treatment [1, 4]. In a survey of Japanese patients with thyroid storm, the leading causes of death included multiorgan failure (24%) and congestive heart failure (21%) [9]. Of note, 84% of patients did achieve complete resolution of their symptoms. Among patients with posttraumatic thyroid storm, the outcome seems to be better than that of thyroid storm of other etiologies.

Thyroid storm should be included in the differential diagnosis of patients with or without known hyperthyroidism who develop rapid onset fever, tachycardia, pulmonary edema, central nervous system manifestations, and distributive shock unresponsive to supportive treatment. Burch and Wartofsky score can be suggestive of thyroid storm; however it is neither specific nor diagnostic. Despite the widely accepted dogma warning against the use of thyroid function testing in the inpatient setting and even more so in the ICU, this is one situation in which thyroid testing is essential.

## 4. Conclusion

This is only the second reported case of thyroid storm secondary to manual strangulation and the first report of strangulation unmasking a previously undiagnosed Graves' disease. This case reveals how strangulation can produce severe thyroid injury and subsequent thyroid storm. Early diagnosis and treatment of thyroid storm can decrease mortality but requires a high degree of clinical suspicion. Treatment is based on blocking thyroid hormone synthesis, preventing release of hormone from thyroid stores, and alleviating peripheral effects of hormone excess. A search for a precipitating cause is critical and should be treated without delay.
